# Discrepant association of serum C-3 epimer of 25-hydroxyvitamin D versus non-epimeric 25-hydroxyvitamin D with serum lipid levels

**DOI:** 10.1186/s12944-016-0333-1

**Published:** 2016-09-15

**Authors:** La-or Chailurkit, Wichai Aekplakorn, Kriangsuk Srijaruskul, Boonsong Ongphiphadhanakul

**Affiliations:** 1 Department of Medicine, Ramathibodi Hospital, Mahidol University, Bangkok, 10400 Thailand; 2 Department of Community Medicine, Ramathibodi Hospital, Mahidol University, Bangkok, 10400 Thailand; 3 Division of Endocrinology and Metabolism, Department of Medicine, Faculty of Medicine Ramathibodi Hospital, Mahidol University, Rama 6th Road, Bangkok, 10400 Thailand

**Keywords:** Epimer, Vitamin D, Serum lipids

## Abstract

**Background:**

Low vitamin D status has been associated with a number of chronic diseases. For dyslipidemia, vitamin D deficiency has been associated with higher low density lipoprotein-cholesterol (LDL-C) in a number of studies, but with inconsistent results in clinical trials. ﻿The purpose of the present study is to explore the relative importance of 3-epi-25-hydroxyvitamin D (25(OH)D) as compared with the non-epimeric form in relation to serum lipid.

**Method:**

This study used data from 1068 randomly selected volunteers in the Thai 4^th^ National Health Examination Survey (NHES IV). Serum 25(OH)D_2_, 25(OH)D_3_, 3-epi-25(OH)D_2_ and 3-epi-25(OH)D_3_ were analyzed by liquid chromatography–tandem mass spectrometry.

**Results:**

There was no association between serum total 25(OH)D and serum LDL-C. However, circulating 3-epi-25(OH)D_3_ was negatively related to serum LDL-C (*r* = −0.077*, P* <0.05), while no such association was found for non-epimeric 25(OH)D_3_ (*r* =0.030, *P* = 0.33). On the other hand, both 3-epi-25(OH)D_3_ (*r* = 0.175, *P* <0.001) and non-epimeric 25(OH)D_3_ (*r* = 0.142, *P* <0.001) were positively related to serum triglyceride (TRIG) levels. In multiple linear regression models with age, gender, body mass index , urban residence, education, hypertension and education as covariates, it was found that 3-epi-25(OH)D_3_ was independently associated with serum LDL-C (beta = −0.12, *P* <0.01), while non-epimeric 25(OH)D_3_ was positively related to LDL-C (beta = 0.13, *P* = 0.002). For TRIG, there were positive association with 3-epi-25(OH)D_3_ (beta = 0.27, *P* <0.001) and negative association with non-epimeric 25(OH)D_3_ (beta = − 0.10, *P* = 0.011) independent of age, gender, urban resident and education.

**Conclusions:**

There is a discrepant association of 25(OH)D levels with serum lipids according to 25(OH)D epimeric forms.

## Background

Vitamin D deficiency is highly prevalent even in tropical countries [1, 2]. Lower vitamin D status has been associated with a number of chronic diseases, including metabolic syndrome and cardiovascular disease [3]. With regard to cardiovascular disease, vitamin D has been shown in both cross-sectional and observational studies to be related to its occurrence [4] and associated mortality. The causality of vitamin D in this regard, as well as the underlying mechanisms involved, are still undetermined. For dyslipidemia, vitamin D deficiency has also been associated with higher levels of low-density lipoprotein cholesterol in a number of studies, but with inconsistent results from clinical trials [5].

The metabolism and action of vitamin D are complex, and this may underlie the incongruence found among studies. C-3 epimers of vitamin D metabolites are present in human circulation [6]. The biological functions of the epimers as compared to native vitamin D are not entirely settled; 3-epi-1α,25-dihydroxyvitamin D (25(OH)_2_D) binds to the vitamin D receptor with less affinity compared with its non-epimeric form [7]. However, 3-epi-1α,25(OH)_2_D suppresses parathyroid hormone to a similar degree to its non-epimeric counterpart [8]. It is unknown at present how C-3 epimers of vitamin D metabolites and lipid metabolism are related. Toward this end, we explored in the present study the relative importance of 3-epi-25(OH)D as compared with the non-epimeric form in their relationship to serum lipid levels.

## Methods

### Study population

This study used a subsample of 1068 participants who were randomly selected by computer-generated random numbers from the Thai 4^th^ National Health Examination Survey (NHES IV), conducted from August 2008 through March 2009 by the National Health Examination Survey Office, Health Systems Research Institute, Thailand, with a sample size of 21,960 Thai individuals. Details of sampling methods have been described previously [9]. Briefly, participants were randomly selects from 21 provinces in four regions of Thailand and the city of Bangkok. The present study drew on a subsample of the data by dividing the total sample into 24 strata based on sex, area of residence (urban or rural for each of the four geographic regions and Bangkok, which was regarded as an urban area only) and age-specific groups (15–29, 30–44, 45–59, 60–69, 70–79 and ≥80 years of age). In each stratum, serum samples were randomly selected using statistical software from each region and Bangkok; ultimately, a total of 1068 Thais were sampled. Demographic data were collected by interviewers. Weight and height were measured by trained field staff using standard procedures. Body mas index (BMI) was calculated as weight in kilograms divided by the square of height in meters. Venous blood samples were obtained from participants in the moring after overnight fast. Fasting plasma glucose was measure on the same day of field data collection. Fasting serum samples were kept at −80 °C before analyses. This study complied with the Declaration of Helsinki. It was also approved by the ethics committee of Ramathibodi Hospital. All subjects gave informed consent prior to the study.

### Definition of diabetes and hypertension

Diabetes was defined as a previous diagnosis of diabetes by a physician and having taken hypoglycemic medication during the prior 2 weeks, or with fasting plasma glucose at the time of the survey ≥126 mg/dL.

Hypertension was defined as mean systolic blood pressure equal to or greather than 140 mm/Hg or mean diastolic blood pressure equal to or greater than 90 mmHg or on medication to lower blood pressure in the past two weeks.

### Serum 25-hydroxyvitamin D (25(OH)D) measurement

All vitamin D metabolites were analyzed by LC-MS/MS with an Agilent 1260 Infinity liquid chromatograph (Agilent Technologies, Waldbronn, Germany) coupled to a QTRAP® 5500 tandem mass spectrometer (AB SCIEX, Foster City, CA, USA) using a MassChrom® 25-OH-Vitamin D3/D2 in serum/plasma reagent kit including a 3-epi-25-OH-Vitamin D3/D2 upgrade diagnostics kit (Chromsystems, Munich, Germany). All analyte values of the calibrator and control were traceable to certified substances and standard reference materials of the National Institute of Standards and Technology. The summation of serum 25(OH)D_3_, 25(OH)D_2_, 3-epi-25(OH)D_3_ and 3-epi-25(OH)D_2_ was used to reflect vitamin D status. The inter-assay and intra-assay coefficients of variation of total serum 25(OH)D level were 6.2 and 9.3 %, respectively.

### Biochemical measurement

Serum cholesterol, low-density lipoprotein cholesterol (LDL-C), high-density lipoprotein cholesterol (HDL-C) and triglyceride (TRIG) levels were analyzed on an automated biochemical analyzer (Dimension RxL; Dade Behring, USA).

### Statistical analyses

Data were expressed as mean ± SD for normal distribution or median and range for non-normal distribution. The relative 3-epimer contribution (%) was used to express the amount of 3-epimer-25(OH)D as a percentage of total 25(OH)D (the sum of 25(OH)D and 3-epi-25(OH)D). The Kolmogorov–Smirnov test was used to test for normality. A logarithmic transformation was performed when the data did not follow a normal distribution. Mann-Whitney U test was used for comparisons of differences between two independent groups. Correlations between two parameters were estimated by using Spearman’s rank correlation coefficient. Multiple linear regression models were used for assessing potential associated factors. A *P* value less than 0.05 was considered statistically significant. All analyses were performed using Stata Statistical Software, Release 12 (StataCorp, College Station, TX, USA).

## Results

Clinical characteristics of the study population are detailed in Table 1. Half of the samples were males and 53 % of all subjects resided in urban. Overll, mean age was 53 year old and mean 25(OH)D was 79.4 nmol/L. More than half (56.4 %) of the subjects had primary education and 35 % had secondary education level or above. 28.8 % of the participants had BMI >=25 kg/m^2^. Hypertension and type 2 diabetes were found in 30.7 and 6.7 % of the subjects, respectively. The relative abundance of 3-epi-25(OH)D compared with total 25(OH)D (relative 3-epimer contribution) ranging from 1.74 to 24.49 %, with a median value of 6.48 %. Subjects with vitamin D insufficiency, as defined by total 25(OH)D levels less than 75 nmol/L, had lower the relative 3-epimer contribution compared to those with vitamin D sufficiency (median 6.34 %, ranged 1.74–14.15 % and median 6.70, ranged 2.56–24.49, repectively) (*P* = 0.001). Table 2 shows the association between serum lipid and 25(OH)D levels. There was no association between serum total 25(OH)D and serum LDL-C. However, circulating 3-epi-25(OH)D_3_ was negatively related to serum LDL-C (*r* = −0.077, *P* <0.05), while no such association was found for non-epimeric 25(OH)D_3_ (*r* = 0.030, *P* = 0.33). On the other hand, both 3-epi-25(OH)D_3_ (*r* = 0.175, *P* <0.001) and non-epimeric 25(OH)D_3_ (*r* = 0.142, *P* <0.001) were positively related to serum TRIG levels. In multiple linear regression models with age, gender, BMI, urban residence, education, hypertension and diabetes as covariates, it was found that 3-epi-25(OH)D_3_ was independently associated with serum LDL-C (beta = −0.12, *P* <0.01), while non-epimeric 25(OH)D_3_ was positively related to LDL-C (beta = 0.13, *P* = 0.002) (Table 3). For TRIG, there were positive association with 3-epi-25(OH)D_3_ (beta = 0.27, *P* < 0.001) and negative association with non-epimeric 25(OH)D_3_, (beta = −0.10, *P* = 0.011) independent of age, gender, urban resident and education.

**Table 1:** Clinical Characteristics of the Study Population (*n* = 1068)

Characteristics	Mean ± SD, or %
Age, years	53.4 ± 22.3
Male	49.8 %
Urban	53.9 %
Weight, kg	57.8 ± 12.7
Education	
< Primary	8.8 %
Primary	56.4 %
Secondary	28.5 %
University	6.5 %
Height, cm	158.0 ± 8.5
BMI, kg/m^2^	23.1 ± 4.6
BMI ≥ 25 kg/m^2^	
No	71.2 %
Yes	28.8 %
Hypertension	
No	69.3 %
Yes	30.7 %
Diabetes	
No	93.3 %
Yes	6.7 %
Cholesterol (mmol/L)	5.3 ± 1.2
LDL-C (mmol/L)	3.4 ± 1.0
HDL-C (mmol/L)	1.2 ± 0.3
Triglycerides (mmol/L)	1.6 ± 1.1
Serum non-epimeric 25(OH)D_3_ (nmol/L)	71.9 ± 20.2
Serum non-epimeric 25(OH)D_2_ (nmol/L)	1.7 ± 2.2
Serum 3-epi-25(OH)D_3_ (nmol/L)	5.5 ± 3.0
Serum 3-epi-25(OH)D_2_ (nmol/L)	0.19 ± 0.07
Total 25(OH)D (nmol/L)	79.4 ± 22.5

**Table 2:** Association between Serum Lipids and 25(OH)D Levels

		Total 25(OH)D	3-epi-25(OH)D_3_	Non-epimeric 25(OH)D_3_	3-epi-25(OH)D_2_	Non-epimeric 25(OH)D_2_
Cholesterol	*r* =	0.061	−0.008	0.064	−0.015	0.001
	*P* =	0.045	0.786	0.036	0.616	0.962
LDL-C	*r* =	0.016	−0.077	0.030	−0.032	−0.062
	*P* =	0.604	0.012	0.328	0.289	0.044
HDL-C	*r* =	−0.063	−0.072	−0.067	0.052	0.035
	*P* =	0.039	0.018	0.030	0.088	0.254
Triglycerides	*r* =	0.161	0.175	0.142	−0.023	0.115
	*P* =	<0.001	<0.001	<0.001	0.456	<0.001

**Table 3:** Multiple Linear Regression According to Predictive Factors and Serum Lipids

Variable	Cholesterol		LDL-C		HDL-C		Triglycerides	
	Beta	*P*-value	Beta	*P*-value	Beta	*P*-value	Beta	*P*-value
Age	0.22	<0.001	0.23	<0.001	−0.08	0.075	0.07	0.1061
Male	−0.17	<0.001	−0.16	<0.001	−0.17	<0.001	0.04	0.211
BMI	0.17	<0.001	0.15	<0.001	−0.22	<0.001	0.20	<0.001
Urban/rural	2.33	0.401	0.02	0.636	0.10	0.002	−0.03	0.344
Education	0.44	0.287	0.04	0.367	0.14	<0.001	−0.05	0.222
Hypertension	7.53	0.024	0.01	0.788	0.03	0.471	0.15	<0.001
Diabetes	0.42	0.940	−0.03	0.281	−0.03	0.394	0.07	0.035
3-epi-25(OH)D_3_	−0.02	0.987	−0.12	0.003	0.05	0.170	0.27	<0.001
Non-epimeric 25(OH)D_3_	0.10	0.035	0.13	0.002	0.01	0.841	−0.10	=0.011

## Discussion

In the present study using a subsample from a nationally representatively survey, we demonstrated that 3-epi-25(OH)D was negatively related to serum LDL-C but positively related to serum TRIG. The association was not likely to be affected by some underlying chronic diseases such as obesity, diabetes and hypertension in particular, as they were controlled in the regression model. Such a relationship was not readily apparent with non-epimeric 25(OH)D levels, or even reversed. With regard to the opposite effect of 3-epi-25(OH)D_3_ vs. its non-epimeric counterpart on serum LDL-C from multiple regression analysis, it is possible that 3-epi-25(OH)D_3_ may exert different influence on LDL-C metabolism from that of 25(OH)D_3._ However, since 3-epi-25(OH)D_3_ and the non-epimeric 25(OH)D_3_ levels were correlated, it is also likely the discrepant finding may be a result of multicollinearilty making the independent effect of each of these metabolites hard to determine in the statistical analysis. For the different direction of association between 3-epi-25(OH)D with LDL-C and TRIG, the underlying reason for the observation is not entirely. However, since hepatic lipase is responsible for the conversion of triglyceride rich very low density lipoprotein to intermediate density lipoprotein and hence LDL, it is conceivable that 3-epi-25(OH)D might reduce the acitivity of hepatic lipase rendering lower LCL-C levels but higher TRIG levels.

Although the causal influence of vitamin D in the determination of serum lipids cannot be concluded from our study, the findings suggest that the effect of vitamin D on lipid metabolism, if any, is likely to be partly dependent on the epimeric forms of vitamin D metabolites. Clinical studies looking at the independent effect of 25(OH)D epimers are scarce. Our finding is in keeping with at least one recent clinical study showing that vitamin D epimers, compared with total or non-epimeric forms of 25(OH), are more related to a number of disease states including type 2 diabetes, rheumatoid arthritis and Alzheimer’s disease [10]. Dyslipidemia, however, was not assessed in the study. With regard to bone health, a more recent study in rodents showed that the C-3α vitamin D epimer increases with higher doses of vitamin D supplementation, without influencing bone mass [11]. In addition, in healthy term infants who received a vitamin D supplement, it was found that 3-epi-25(OH)D_3_ was not associated with body composition [12].

Although the role of LDL-C in atherosclerosis is well established, the role of raised TRIG in cardiovascular disease is controversial. The adverse effect of hypertriglyceridemia on cardiovascular disease, if any, is more likely to occur with serum triglycerides in the mild to middle range, since at higher levels the lipoproteins could be too large to enter the arterial intima [13, 14]. However, genetic studies, including those using the Mendelian randomization approach, have suggested that triglyceride-rich lipoproteins are a causal risk factor of cardiovascular disease [15, 16]. The effect of vitamin D on cardiovascular disease and mortality has been suggested. A recent meta-analysis showed the influence of vitamin D_3_ on mortality, while vitamin D_2_ did not appear to have any effect [17]. A more recent systematic review also demonstrated the effect of vitamin D on mortality associated with various causes, including those related to cardiovascular disease and cancer [18]. In addition, Ciccone et al., has found a significant correlation of vitamin D deficiency with mortality for major cardiovascular events such as heart failure, myocardial infarction, sudden cardiac death, stroke, atrial fibrillation, and peripheral vascular disease [19]. The causal role of vitamin D, however, in cardiovascular diseases and others is controversial [20]. Likewise, the causal relationship between vitamin D epimers and dyslipidemia as well as other disease states is unclear. Nevertheless, there are indications of biological possibility. The epimer 3-epi-25(OH)D_3_ can undergo 1α-hydroxylation to form 3-epi-1,25(OH)_2_D_3_, which can bind to the vitamin D receptor and activate gene transcription [21]. It appears that 3-epi-1,25(OH)_2_D_3_ is nearly potent as 1,25(OH)_2_D_3_ in suppressing parathyroid hormone secretion but has significantly reduced calcemic effects [8, 22].

The observed correlation between 3-epi-25(OH)D and serum lipids in the present study may be part of the underlying basis for the association between vitamin D status and cardiovascular health. A small study found an association between vascular health indices and serum total and free 25(OH)D in adolescents [23].

Despite the promising effect of vitamin D on serum lipids, interventional trials with vitamin D have generally failed to demonstrate a causal association. A number of previous studies have investigated the relationship between vitamin D status and serum lipids. For example, calcium/vitamin supplementation was found to have a favorable effect on serum lipids in the Women’s Health Initiative study [24]. A small clinical trial using vitamin D-fortified skimmed milk found a reduction in blood pressure but no beneficial effect of the intervention on serum lipids [25]. However, blood pressure reduction was not found in another study of vitamin D supplementation [26]. Besides the probable difference in study design and study populations, it is likely that between-subject differences in the proportion of the epimeric versus non-epimeric forms of vitamin D after supplementation may partly be accountable for the inconsistent results. Further clinical trials looking at the effect of vitamin D should probably include the assessment of vitamin D epimers.

A number of limitations are present in this study. The study was cross-sectional in nature and the causative role of vitamin D epimers, if any, could not be readily determined. Moreover, although none of the subjects in the present study were taking lipid-lowering agents, data regarding other concurrent medications or supplements, dietary intake of vitamin D and food consumption which may influence serum lipids or vitamin D status and the association were not available. Further studies controlling for such variables are therefore warranted.

## Conclusion

This is the first report of the discrepant association of 25(OH)D levels with serum lipids according to 25(OH)D epimeric forms. The relative amount of 3-epi- versus non-epimeric 25(OH)D after vitamin D supplementation may be attributable to the discrepant findings among clinical trials.

## Abbreviations

LDL-C: Low density lipoprotein-cholesterol
HDL-C: High-density lipoprotein cholesterol
TRIG: Triglyceride
25(OH)D: 25-hydroxyvitamin D
BMI: Body mass index
